# Does community social capital buffer the relationship between educational disadvantage and cognitive impairment? A multilevel analysis in Japan

**DOI:** 10.1186/s12889-019-7803-0

**Published:** 2019-11-01

**Authors:** Hiroshi Murayama, Fumiko Miyamae, Chiaki Ura, Naoko Sakuma, Mika Sugiyama, Hiroki Inagaki, Tsuyoshi Okamura, Shuichi Awata

**Affiliations:** 1 Institute of Gerontology, The University of Tokyo, 7-3-1 Hongo, Bunkyo-ku, Tokyo, 113-8656 Japan; 2 Research Team for Promoting Independence and Mental Health, Tokyo Metropolitan Institute of Gerontology, 35-2 Sakae-cho, Itabashi-ku, Tokyo, 173-0015 Japan

**Keywords:** Buffering effect, Cognitive impairment, Japan, Multilevel analysis, Older people, Social capital

## Abstract

**Background:**

This study explored the relationship between community social capital and cognitive impairment, with a focus on the buffering role of community social capital in the association between educational disadvantage and cognitive impairment in community-dwelling older adults in Japan.

**Methods:**

We used data from two population-based, cross-sectional surveys targeting people aged ≥65 years in a suburban city of the Tokyo metropolitan area (*n* = 897; 49.8% men; average age = 74.4 years). Social capital included social support (emotional and instrumental support) and the strength of social networks (neighborly ties). To create district-level social capital indicators, we aggregated individual responses on social capital within each district. The Mini-Mental State Examination, Japanese version was used for the assessment of cognitive function.

**Results:**

Using multilevel logistic regression analysis, we found that lower amounts of district-level emotional and instrumental support were associated with a greater likelihood of cognitive impairment among men. For women, district-level emotional support was associated with a greater likelihood of cognitive impairment. Additionally, a strong district-level social network buffered the relationship between low education and cognitive impairment in both sexes.

**Conclusions:**

Community social capital appears to have a protective role in determining cognitive function in old age. Our findings may facilitate the development of new community-based strategies to combat dementia.

## Background

To date, many studies have explored risk factors for cognitive decline, including physical, medical, nutritional, economic, behavioral, and genetic factors [1, 2]. In addition to links between cognitive decline and these individual factors, the associations between neighborhood environmental factors and cognitive function have been widely recognized [3]. In particular, the characteristics of built environments have been examined in terms of their effect on cognitive function [4, 5]. However, it has been proposed that the qualities of social environments, as well as built environments, influence cognition, with social capital as a potentially important factor [3].

Public health researchers, particularly those in the field of social epidemiology, have explored the association between social capital and health. Kawachi and Berkman [6] defined social capital as resources that are available to individuals as a result of their membership in a network or a group. Social capital is generally classified as “individual-level” or “group-level.” At the individual level, social capital refers to resources embedded within an individual’s social networks. In contrast, at the group level, social capital represents the resources available to members of communities.

Several articles have examined the relationship between individual-level social capital and cognitive functioning. Meta-analyses have reported that a higher level of social relationships were inversely associated with cognitive decline [7] and with dementia incidence [8]. However, few studies have examined the link between community (i.e., group-level) social capital and cognitive function. Hikichi et al. [9] reported that community-level informal socializing and social participation lowered the risk of cognitive decline in the aftermath of the Great East Japan Earthquake in 2011. Murayama et al. [10] showed that denser community-level social networks were related to a lower likelihood of subjective dementia symptoms in older people (particularly among women) in a Tokyo urban area. To our knowledge, to date, these two studies are the only research to explore the contextual association of social capital with cognitive outcomes.

Population aging is a global issue that will unavoidably lead to an increased prevalence of cognitive decline and dementia. As mentioned above, cognitive decline is influenced by not only individual factors but also by neighborhood environmental factors. To develop community-based strategies for delaying/preventing cognitive decline and the onset of dementia, more evidence is needed regarding the relationship between community social capital and cognitive functioning. Hikichi et al.’s [9] study was conducted in an earthquake-stricken area, which was characterized by a particular set of conditions. Murayama et al. [10] administered their study among a general community-dwelling older population, but their research was conducted in the Tokyo urban area—one of the most overpopulated areas in the world. Because the historical and geographical backgrounds of communities affect the form of community social capital [11], findings on community social capital and cognitive decline in various types of settings should be accumulated.

Existing studies also used specific forms of outcome measurements. In particular, they assessed cognitive function using a standardized in-home assessment scale that is part of the Japanese long-term care insurance scheme [12] and a self-administered dementia checklist [13, 14]. However, these are not necessarily optimal measures of cognitive function or dementia. The Mini-Mental State Examination (MMSE) is one of the most commonly used tools for assessing cognitive function and screening for dementia in worldwide clinical and research settings. Thus, using the MMSE as the outcome measure regarding the effect of community social capital on cognitive functioning would likely increase the strength and generalizability of the research findings.

In addition to the main effect of community social capital on health outcomes, previous studies have suggested that community social capital can also act as a buffer against social disadvantages [15]. For example, Fone et al. [16] showed that area-level social cohesion appears to buffer the effect of area-level income deprivation on mental illness. Takagi et al. [17] revealed that higher area-level social trust among neighbors and higher levels of social participation buffered the deleterious effects of social distance (i.e., sociodemographic differences between individuals and their neighbors) on depressive mood in older adults. Moreover, Hikichi et al. [9] reported that community-level informal socializing and social participation could ameliorate the adverse effect of earthquake-induced housing damage on cognitive decline. New information about its buffering role may contribute to identifying the mechanisms by which community social capital affects individual health; however, evidence is still sparse, particularly regarding factors that mediate cognitive function.

Given these considerations, this study aimed to explore the relationship between community social capital and cognitive impairment in community-dwelling older adults in Japan. In addition, educational disadvantage in early life (i.e., having only a few years of education) is a well-known risk factor for cognitive decline/impairment and dementia onset [18–20]. Therefore, we also examined the buffering role of community social capital in the association between educational disadvantage and cognitive impairment.

## Methods

### Study population

We used data derived from population-based, cross-sectional surveys of community residents aged ≥65 years living in a suburban area of metropolitan Tokyo. The study was conducted in Machida, a city located in the southern part of Tokyo that is known for its strong efforts and interventions in dementia care and prevention. As of June 1, 2013, the city’s total population was 425,762 (209,693 men and 216,069 women), and 23.3% were aged ≥65 years [21]. Machida city had 157 district areas (called *chou-chou* in Japanese) in 2013.

We administered two original surveys (a mail-in questionnaire survey and a home-interview survey), mainly in the northeastern part of the city where there was a hospital specializing in the treatment of dementia. After discussions with Machida municipal government staff, to avoid an area-level sampling bias, we selected 22 district areas on the basis of their land use characteristics (e.g., houses, public and private apartments, or farming), population size, and proportion of people aged ≥65 years.

First, we conducted a mail-in questionnaire survey for all older residents of the 22 selected district areas in June and July of 2013 to assess sociodemographic characteristics, social relationships, and health behaviors (*n* = 7682). In total, 6932 questionnaires were returned (response rate: 90.2%), and all were regarded as valid responses.

Second, we assessed cognitive impairment using the MMSE, Japanese version (MMSE-J) [22] and health conditions via a home-interview survey in November and December of 2013. Among older individuals living in the 22 district areas (the same sample as in the first survey; *n* = 7682), we randomly selected a total of 3000 people after stratifying the group by age and sex. Among these residents, 2786 were eligible, and we asked them by mail to participate in the home-visit survey. As a result, 1341 people were then interviewed by trained registered nurses (response rate: 48.1%). After we excluded 22 individuals who did not complete the MMSE-J, the responses of 1319 people were regarded as valid.

Because social capital was measured in the mail-in questionnaire survey for only 17 of the 22 selected district areas, this study included data collected from individuals living in these 17 district areas (number of valid responses: 4649 for the mail-in questionnaire survey and 897 for the home-interview survey). All 897 respondents in the home-interview survey had also responded to the mail-in questionnaire survey, and their data from the two surveys were combined for use in the analysis.

The study protocol was approved by the Ethical Committee of Tokyo Metropolitan Institute of Gerontology. Participants were informed about the study purpose, method, survey items, and merits of participation. The return of the questionnaire was viewed as consent to participate in the mail-in questionnaire the survey. Written consent was received from all participants of the home-interview survey.

### Individual-level measures

#### Cognitive function

Cognitive function was assessed using the MMSE-J in the home-interview survey. The MMSE is one of the most widely used tools to assess cognitive function worldwide, making it ideal to increase the strength and generalizability of the results. To ensure valid and reliable interviewing and testing, the trained registered nurses who conducted the interviews and administered the MMSE-J completed a 2-day training session with geriatric psychiatrists and psychologists. Details regarding the training sessions are available elsewhere [23]. MMSE-J scores range from 0 to 30, and higher scores indicate greater cognitive function. We adopted a cut-off value of 23/24, meaning that a score of ≤23 indicated cognitive impairment [22].

#### Social capital

In this study, we assessed two dimensions of social capital (cognitive and structural) because this is a popular distinction in social capital [6, 24]. We measured social support and social network as indicators of the cognitive and structural dimensions of social capital, respectively. First, two questions were used to measure social support; one was for emotional support (“How many friends do you have with whom you can talk about private affairs?”), and the other was for instrumental support (“How many friends do you have whom you can ask for help?”). Respondents answered these items on a five-point scale (five or more persons [= 1], three to four persons [= 2], two persons [= 3], one person [= 4], or none [= 5]). Second, we assessed social networks by examining neighborly ties. Specifically, we included one item, rated using a four-point scale: “How is your relationship with your neighbors?” (I often talk with neighbors about my problems [= 1], I only make small talk with my neighbors [= 2], I only greet my neighbors [= 3], or I am not friendly with my neighbors at all [= 4]).

#### Sociodemographic characteristics, health behaviors, and health conditions

We asked participants to report their age, sex, marital status (married or unmarried), years of education, annual income (< 2.0 million yen, 2.0–4.9 million yen, or ≥ 5.0 million yen), and current smoking status (smoker or non-smoker) in the mail-in questionnaire survey. The number of years of education was dichotomized in the analysis (≤ 9 years or ≥ 10 years). In addition, a trained registered nurse assessed information regarding comorbidity in the home-interview survey, particularly with respect to hypertension, heart disease, stroke, diabetes, cancer, and respiratory disease. We calculated the number of conditions for which participants were currently receiving treatment and divided them into three categories accordingly (0, 1, or ≥ 2).

### District-level measures

Because participants were nested within 17 districts, to capture district-level social capital, we aggregated individual responses for items concerning social capital in the district in which the participants resided. To increase the validity of the indicators, we used all of the individual responses received for the mail-in questionnaire survey (*n* = 4649) for this part of the analysis. We calculated the proportions of (i) people who reported that they had no friends or one friend with whom they could talk about private affairs, (ii) people who reported that they had no friends or one friend whom they could ask for help, and (iii) people who answered “I only greet my neighbors” or “I am not friendly with my neighbors at all” (Categories 3 or 4) within the district as indicators of district-level emotional support, instrumental support, and social network, respectively.

In addition, the proportion of people aged ≥65 years and population density (persons/km^2^) of the districts were included in the analysis. We obtained information on the total population size, the number of people aged ≥65 years, and the area of each district (as of November 1, 2013) from the official website of Machida city [21] to calculate the proportion of people aged ≥65 years and population density. The population density was used to consider the effects of the urbanization of the district.

### Statistical analysis

To examine the association between district-level social capital and cognitive impairment in individual participants, we fitted the data using a multilevel logistic regression model that included a random intercept. The estimation was performed using the full maximum likelihood procedure. Because district-level social capital was aggregated from individual responses, there was a chance that individual-level indicators of social capital could act as confounders concerning the association between district-level social capital and cognitive impairment [25]. Therefore, both individual- and district-level social capital indicators were simultaneously added into the model. To understand the buffering role of community social capital in the association between educational disadvantage and cognitive impairment, we tested the interaction between educational level and each indicator of community social capital on the outcome.

We adopted the following modeling strategy. In Model 1, each individual-level and district-level social capital indicator (i.e., emotional support, instrumental support, and social network) was included separately. In Model 2, a cross-level interaction between education and each variable of district-level social capital was added to Model 1. The results of the fixed effects are expressed as odds ratios (ORs) with 95% confidence intervals (CIs). All analyses were stratified by sex, using HLM 7.03 (Scientific Software International, Inc., Skokie, IL, USA).

## Results

The characteristics of individual participants are shown in Table 1. Among the 897 participants, 49.8% were men, and the average age was 74.4 years. With regard to social support, 32.4% had no friends with whom they could talk about private affairs, and 48.1% had no friends whom they could ask for help. In terms of social network, 7.4% had no neighborly ties. These proportions were higher for men than for women. The proportion of people with cognitive impairment (MMSE-J ≤ 23) was 10.9%, and this was slightly lower for women than for men (11.2% vs. 10.7%).

**Table 1:** Participant characteristics

	Total (*n* = 897)	Men (*n* = 447)	Women (*n* = 450)
Age (years)	74.4 ± 6.3	74.4 ± 6.2	74.5 ± 6.4
Unmarried	31.1	18.4	47.7
Years of education	12.5 ± 2.9	13.4 ± 3.1	11.6 ± 2.4
≤ 9 years	23.5	19.7	27.2
Annual income			
≥ 5.0 million yen	12.0	15.1	8.9
2.0–4.9 million yen	62.1	69.4	54.7
< 2.0 million yen	25.8	15.5	36.5
Smoker	11.6	17.5	5.8
Number of chronic diseases			
None	41.7	38.1	45.3
1	16.8	34.5	39.1
≥ 2	21.4	27.4	15.6
Number of friends with whom they could talk about private affairs (emotional support)	3.5 ± 1.4	3.6 ± 1.4	3.3 ± 1.3
Five or more persons [= 1]	9.8	9.4	10.3
Three to four persons [= 2]	16.7	14.9	18.5
Two persons [= 3]	20.8	17.7	24.0
One person [= 4]	20.3	18.3	22.1
None [= 5]	32.4	39.7	25.1
Number of friends whom they could ask for help (instrumental support)	3.9 ± 1.2	4.0 ± 1.3	3.9 ± 1.2
Five or more persons [= 1]	4.3	4.4	4.2
Three to four persons [= 2]	12.2	13.2	11.2
Two persons [= 3]	17.7	14.6	20.8
One person [= 4]	17.7	13.9	21.5
None [= 5]	48.1	53.9	42.3
Neighborly ties (social network)	2.3 ± 0.8	2.5 ± 0.8	2.0 ± 0.8
I often talk with neighbors about my problems [= 1]	16.7	8.0	25.3
I only make small talk with my neighbors [= 2]	46.6	41.5	51.7
I only greet my neighbors [= 3]	29.4	41.9	16.9
I am not friendly with my neighbors at all [= 4]	7.4	8.7	6.1
Cognitive impairment (MMSE-J ≤ 23)	10.9	11.2	10.7

Values represent % or means ± standard deviations

*MMSE-J* Mini-Mental State Examination, Japanese version

We excluded 3752 participants when combining the data from the two surveys because they did not participate in the home-interview survey. The proportion of men was higher in the analytic sample (*n* = 897) than in the excluded sample (*n* = 3752; 49.8% vs. 44.5%; *p* = 0.005), and the average age was higher in the analytic sample than in the excluded sample (74.4 years vs. 73.7 years; *p* = 0.002); however, there were no differences in the number of years of education (12.5 years for both samples; *p* = 0.992).

Table 2 presents the characteristics of the study districts. On average, 19.7% of people were aged ≥65 years (range: 11.9–33.4%). The proportions of people who had no friends or one friend with whom they could talk about private affairs within the district, people who had no friends or one friend whom they could ask for help within the district, and people who answered “I only greet my neighbors” or “I am not friendly with my neighbors at all” within the district were 51.4, 65.3, and 37.8%, respectively.

**Table 2:** Characteristics of the study districts (*n* = 17)

	Mean ± SD	Median	Min–Max
Analytic sample			
Number of respondents to the mail-in questionnaire survey	273.5 ± 171.7	267	20–706
Number of respondents to the home-interview survey	53.6 ± 31.0	53	4–116
Demographic factors			
Population size (persons)	1650.1 ± 750.5	1565	142–3351
% of people aged ≥65 years	19.7 ± 6.0	17.4	11.9–33.4
Population density (persons/km^2^)	8082.5 ± 2575.9	8074.6	1538.8–11,535.7
Social capital			
Low emotional support			
% of people who had no friends or one friend with whom they could talk about private affairs in the district	51.4 ± 4.9	49.5	45.0–60.0
Low instrumental support			
% of people who had no friends or one friend who they could ask for help in the district	65.3 ± 3.9	63.8	60.7–72.7
Weak social network			
% of people who answered “I only greet my neighbors” or “I am not friendly with my neighbors at all” in the district	37.8 ± 5.0	37.6	25.7–50.0

*SD* standard deviation

The multilevel logistic regression analysis results are shown in Table 3 (men) and Table 4 (women). Because the proportion of female smokers was small, the model that included smoking status as an independent variable did not converge. Therefore, we excluded smoking status from the analysis for women. Among men, after controlling for individual-level and district-level covariates, lower levels of district-level emotional support and instrumental support were significantly associated with a higher likelihood of cognitive impairment in Model 1 (OR [95% CI]: 1.67 [1.14–2.44] for emotional support and 2.09 [1.49–2.94] for instrumental support). Among women, lower emotional support at the district level was significantly associated with cognitive impairment (1.58 [1.00–2.49]).

**Table 3:** Associations between district-level social capital and cognitive impairment in men

	Model 1			Model 2		
	OR (95% CI)	OR (95% CI)	OR (95% CI)	OR (95% CI)	OR (95% CI)	OR (95% CI)
Individual level						
Older (every 10-year increase in age)	3.89 (2.00–7.59)^***^	3.95 (2.10–7.41)^***^	3.60 (2.00–6.51)^***^	3.97 (1.96–8.03)^***^	3.89 (2.04–7.42)^***^	3.67 (1.96–6.87)^***^
Unmarried	2.62 (1.36–5.07)^**^	2.66 (1.41–5.03)^**^	2.76 (1.38–5.51)^**^	2.70 (1.44–5.04)^**^	2.71 (1.47–5.03)^**^	3.11 (1.47–6.56)^**^
Low education						
≤ 9 years of schooling	8.76 (3.22–23.88)^***^	7.90 (2.94–21.23)^***^	7.78 (2.68–22.57)^***^	11.61 (2.74–49.30)^**^	10.39 (2.78–38.92)^**^	11.06 (3.33–36.73)^***^
Annual income (ref.: ≥ 5.0 million yen)						
2.0–4.9 million yen	0.79 (0.24–2.59)	0.82 (0.28–2.40)	0.81 (0.28–2.31)	0.66 (0.21–2.12)	0.67 (0.23–1.94)	0.69 (0.23–2.11)
< 2.0 million yen	1.74 (0.33–9.05)	1.83 (0.38–8.93)	1.87 (0.40–8.79)	1.71 (0.36–8.21)	1.77 (0.38–8.20)	1.93 (0.43–8.69)
Smoker	3.33 (1.72–6.44)^***^	2.98 (1.47–6.05)^**^	2.47 (1.27–4.79)^**^	3.40 (1.72–6.73)^***^	3.02 (1.46–6.24)^**^	2.71 (1.41–5.20)^**^
Number of chronic diseases (ref.: none)						
1	1.08 (0.47–2.52)	0.99 (0.44–2.27)	1.19 (0.56–2.52)	0.94 (0.41–2.12)	0.91 (0.41–2.04)	1.14 (0.54–2.38)
≥ 2	0.59 (0.16–2.22)	0.57 (0.14–2.34)	0.52 (0.14–2.00)	0.46 (0.10–2.16)	0.49 (0.10–2.33)	0.47 (0.11–2.08)
Smaller number of friends with whom they can talk about private affairs	0.87 (0.70–1.07)			0.87 (0.70–1.08)		
Smaller number of friends whom they can ask for help		0.91 (0.71–1.17)			0.92 (0.70–1.20)	
Weaker neighborly ties			0.77 (0.41–1.45)			0.73 (0.37–1.41)
District level						
% of people aged ≥65 years in the district (every 10% increase)	1.02 (0.64–1.61)	1.17 (0.75–1.82)	0.92 (0.54–1.56)	1.05 (0.64–1.71)	1.16 (0.74–1.82)	0.90 (0.52–1.58)
Population density in the district (every 1000 persons/km^2^ increase)	1.02 (0.93–1.11)	1.00 (0.91–1.10)	1.10 (0.99–1.23)	1.00 (0.91–1.11)	1.00 (0.91–1.10)	1.10 (0.97–1.24)
Lower emotional support						
% of people who had no friends or one friend with whom they could talk about private affairs in the district (every 10% increase)	1.67 (1.14–2.44)^*^			2.19 (1.16–4.11)^*^		
Lower instrumental support						
% of people who had no friends or one friend whom they could ask for help in the district (every 10% increase)		2.09 (1.49–2.94)^***^			2.31 (1.20–4.45)^*^	
Weaker social network						
% of people who answered “I only greet my neighbors” or “I am not friendly with my neighbors at all” in the district (every 10% increase)			1.50 (0.98–2.27)			1.03 (0.59–1.80)
Cross-level interactions						
Low educational level × lower district-level emotional support				0.25 (0.03–2.15)		
Low educational level × lower district-level instrumental support					0.68 (0.05–10.05)	
Low educational level × weaker district-level social network						5.06 (1.37–18.62)^*^

*CI* confidence interval, *OR* odds ratio

^***^ *p* < 0.001. ^**^ *p* < 0.01. ^*^ *p* < 0.05

**Table 4:** Associations between district-level social capital and cognitive impairment in women

	Model 1			Model 2		
	OR (95% CI)	OR (95% CI)	OR (95% CI)	OR (95% CI)	OR (95% CI)	OR (95% CI)
Individual level						
Older (every 10-year increase in age)	4.15 (2.64–6.52)^***^	4.39 (2.72–7.09)^***^	4.31 (2.79–6.66)^***^	3.89 (2.48–6.10)^***^	4.24 (2.62–6.87)^***^	4.13 (2.60–6.55)^***^
Unmarried	0.58 (0.26–1.33)	0.59 (0.26–1.35)	0.52 (0.22–1.21)	0.57 (0.25–1.29)	0.56 (0.24–1.27)	0.53 (0.23–1.23)
Low education						
≤ 9 years of schooling	2.19 (1.03–4.64)^*^	2.30 (1.12–4.73)^*^	2.38 (1.31–4.33)^**^	2.23 (0.98–5.10)	2.31 (1.13–4.73)^*^	2.68 (1.26–5.72)^*^
Annual income (ref.: ≥ 5.0 million yen)						
2.0–4.9 million yen	1.62 (0.49–5.31)	1.69 (0.52–5.50)	1.51 (0.50–4.57)	1.93 (0.66–5.61)	2.05 (0.71–5.93)	1.59 (0.53–4.77)
< 2.0 million yen	1.53 (0.56–4.20)	1.69 (0.63–4.56)	1.23 (0.45–3.35)	1.78 (0.69–4.64)	2.01 (0.79–5.09)	1.20 (0.44–3.26)
Number of chronic diseases (ref.: none)						
1	0.47 (0.25–0.91)^*^	0.45 (0.23–0.87)^*^	0.47 (0.26–0.83)^*^	0.52 (0.26–1.05)	0.48 (0.25–0.94)^*^	0.50 (0.27–0.92)^*^
≥ 2	0.44 (0.14–1.41)	0.40 (0.12–1.34)	0.40 (0.12–1.32)	0.44 (0.13–1.89)	0.39 (0.11–1.31)	0.43 (0.12–1.48)
Smaller number of friends with whom they could talk about private affairs	1.22 (0.90–1.65)			1.21 (0.89–1.64)		
Smaller number of friends whom they could ask for help		1.10 (0.89–1.36)			1.08 (0.88–1.34)	
Weaker neighborly ties			1.63 (1.26–2.12)^***^			1.64 (1.25–2.15)^***^
District level						
% of people aged ≥65 years in the district (every 10% increase)	0.82 (0.52–1.28)	0.89 (0.55–1.42)	0.91 (0.56–1.49)	0.79 (0.52–1.20)	0.86 (0.54–1.38)	0.90 (0.57–1.44)
Population density in the district (every 1000 persons/km^2^ increase)	1.00 (0.89–1.21)	1.03 (0.93–1.13)	1.04 (0.95–1.15)	1.00 (0.91–1.11)	1.03 (0.94–1.13)	1.07 (0.97–1.17)
Lower emotional support						
% of people who had no friends or one friend with whom they could talk about private affairs in the district (every 10% increase)	1.58 (1.00–2.49)^*^			1.70 (1.01–2.87)^*^		
Lower instrumental support						
% of people who had no friends or one friend whom they could ask for help in the district (every 10% increase)		1.35 (0.69–2.66)			1.61 (0.78–3.30)	
Weaker social network						
% of people who answered “I only greet my neighbors” or “I am not friendly with my neighbors at all” in the district (every 10% increase)			1.13 (0.70–1.82)			1.02 (0.67–1.55)
Cross-level interactions						
Low educational level × lower district-level emotional support				0.48 (0.12–1.89)		
Low educational level × lower district-level instrumental support					0.21 (0.03–1.42)	
Low educational level × weaker district-level social network						3.18 (1.02–10.30)^*^

*CI* confidence interval. *OR* odds ratio

^***^ *p* < 0.001. ^**^ *p* < 0.01. ^*^ *p* < 0.05

**Fig. 1:**
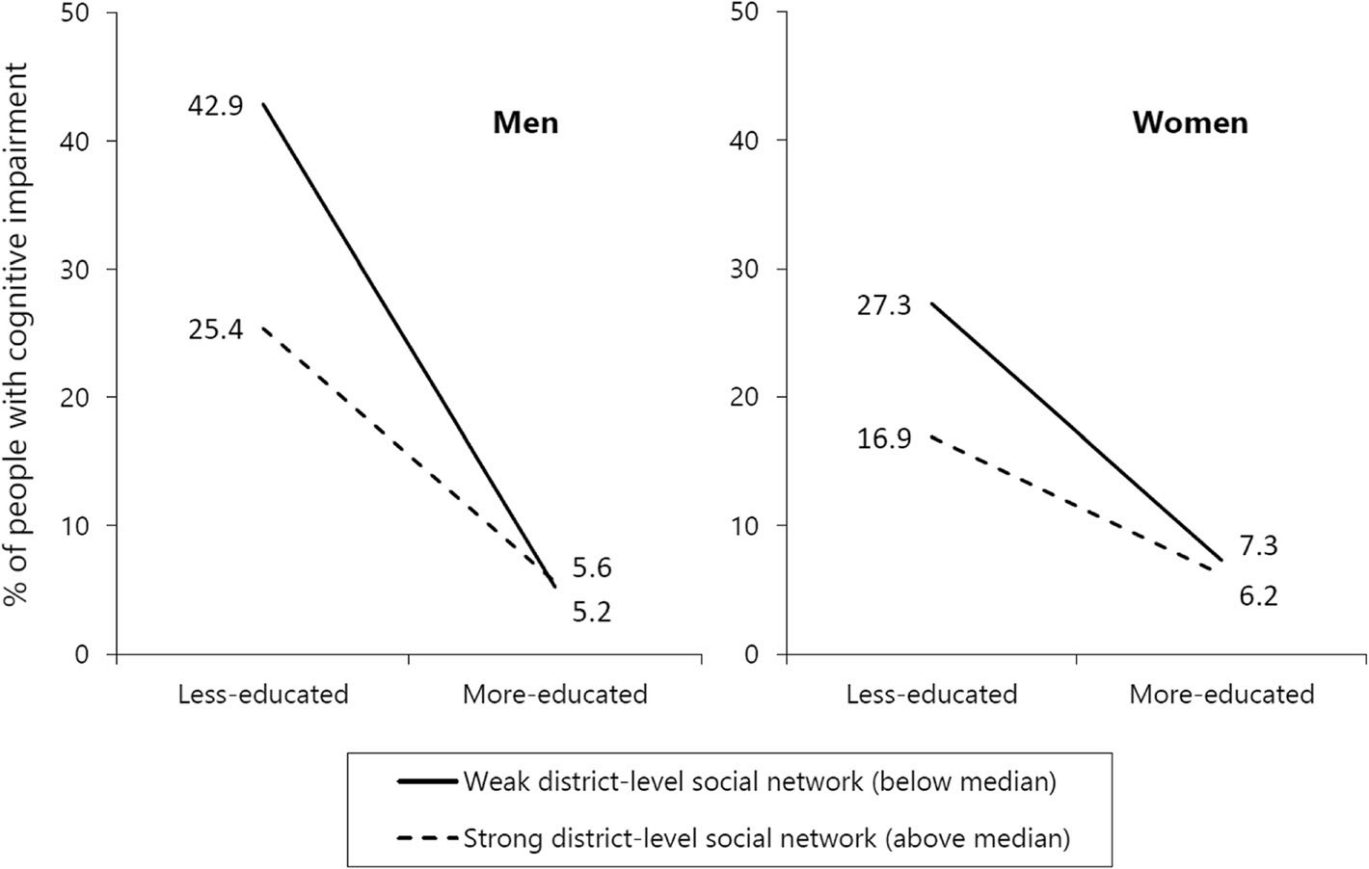
Proportion of people with cognitive impairment by educational level and district-level social network (unconditional model). For ease of presentation, district-level social network was dichotomized into strong (above median) and weak (below median) categories

Next, to determine whether community social capital had a buffering role in the association between educational disadvantage and cognitive impairment, we added a cross-level interaction between education and community social capital in Model 2. We observed a significant interaction between low educational level and weaker district-level social networks in both men and women (5.06 [1.37–18.62] for men and 3.18 [1.02–10.30] for women). Figure 1 illustrates these interactions. For ease of presentation, we divided district-level social networks into two categories (strong and weak) at the median value. Among highly educated participants, we found no difference in the proportion of cognitive impairment between individuals with strong and weak social networks at the district level. However, among less-educated participants, the proportion with cognitive impairment was lower in the districts with strong social networks than in the districts with weak social networks. This trend was more apparent for men than for women.

## Discussion

Using data from two population-based surveys of community-dwelling older adults in Japan, this study explored the association between community social capital and cognitive impairment (as assessed by the MMSE-J). We also investigated the buffering role of community social capital in the relationship between educational disadvantage and cognitive impairment. To the best of our knowledge, this is the first study to examine the link between community social capital and cognitive impairment as measured using the MMSE. Because the MMSE is used worldwide in clinical and research settings to measure global cognitive ability, we anticipate that our results, obtained using the MMSE-J, will expand upon previous findings and provide new insight in the field of social capital research. Such findings may be particularly useful in guiding policymakers as they develop community-based strategies to address dementia.

Lower levels of district-level emotional and instrumental support were associated with a higher likelihood of cognitive impairment in men, and this was also the case for district-level emotional support in women. The direct relationship between district-level social support and cognitive impairment may operate via a number of pathways. People living in communities with strong social support systems might find it easier to cope with daily stress [6], thus suppressing depressive mood [26]. Because depression is known to be a risk factor for cognitive decline [27], strong community social support could be associated with a lower likelihood of cognitive impairment among residents.

Improved access to local services and amenities, which may be a characteristic of strong community social support systems [28], could also decrease the likelihood of cognitive impairment. Close supportive connections within a neighborhood have been found to effectively facilitate the sharing of private personal needs. Knowledge about these needs is useful in developing necessary services and amenities in response to needs in communities and might improve local access [6]. Additionally, improving access to services and amenities may also promote social participation among residents, which could also result in the delay or prevention of cognitive impairment [29].

Social isolation is a risk factor for dementia and cognitive impairment [20]. Previous research has reported that fostering intergenerational helping networks in the community can prevent social isolation/loneliness among older adults [30]. Therefore, the association between greater district-level social support and a lower likelihood of cognitive impairment may have been generated through reduced social isolation in the community.

Murayama et al. [10] reported that community-dwelling older adults in urban Tokyo who lived in communities with denser neighborly ties, which are a structural aspect of community social capital, were less likely to have subjective dementia symptoms. They also found that this was particularly the case among women. However, in the present study, which assessed community-dwelling older adults in a suburban Tokyo area, the strength of the social network in the district in which participants resided was not significantly associated with cognitive impairment for either men or women. This gap in findings might have been caused by differences in the outcome measurement and study design (e.g., sample size), but the gap may also be explained by differences in the influence of different types of neighborhood networks on the health of individuals. Neighborhood connections are known to be much denser in rural and suburban areas compared with urban areas in Japan [31]. Thus, community neighborhood connections might have a relatively smaller impact on health status among individuals living in suburban areas (i.e., the setting of the present study) compared with those in urban areas.

In addition to the direct association between community-based cognitive social capital and cognitive impairment (i.e., the main effect), we found a significant cross-level interaction between low educational level and district-level social network on cognitive impairment for both sexes. Specifically, less-educated people living in communities with strong structural social capital were less likely to exhibit signs of cognitive impairment than were less-educated people living in communities with weak structural social capital. One possible explanation for this association is the accumulation of deviant health behaviors. It is well-established that less-educated people are more likely to adopt unhealthy behaviors (e.g., smoking, heavy drinking, and physical inactivity) [32–34], which are known to increase the risk of cognitive decline [20, 35]. However, less-educated participants living in districts with strong social networks might be able to quickly and easily obtain information regarding dementia prevention (e.g., effective health behaviors and disease control) via tight-knit community networks. Indeed, according to the diffusion of innovation theory, ideas and innovations propagate more quickly in such communities (generally called “social contagion”) [36].

Moreover, deviant health behaviors in less-educated people could be inhibited through informal social control. As those living in communities with higher social capital tend to work harder to maintain social order, they might step in to intervene via close connections in the community when they witness others engaging in deviant behaviors [6]. In addition, social learning theory posits that observing and imitating others is an important factor for behavioral acquisition [37]. In communities with stronger social networks, less-educated people might have more chances to observe and imitate others’ healthy behaviors related to dementia prevention, and thus be more likely to adopt these behaviors successfully. However, because this study did not assess items on health behaviors other than smoking status (e.g., physical activity and alcohol consumption), we were not able to examine this hypothesis clearly. Future studies should consider this point.

Lower education is a known risk factor for dementia onset [18–20]. Our study showed that community social capital (particularly community social network) could be a modifier of the relationship between education and cognitive function. This implies that, in planning an intervention to reduce the educational disparity with the aim of preventing cognitive decline or dementia, we should treat the level of social capital in the intervention area as a segmenting variable.

There are several limitations to the present study. First, the size of the analyzed district was small. Future studies including a greater number of districts are necessary to obtain more robust results. Second, the response rate for the home-interview survey was not high (approximately 50%), which indicates that selection bias may have occurred, possibly leading to an overestimation of the examined associations. Indeed, the analytic sample (*n* = 897) included more men and older participants compared with the excluded sample (*n* = 3752). Third, we aggregated the individual responses of older people within districts to create district-level social capital variables. More genuine indicators of social capital should be used (e.g., including young and middle-aged populations) in future work. Fourth, the target community was limited to a suburban city in the Tokyo metropolitan area. As the historical and geographical contexts of communities are known to influence community social capital [11], we should be careful in generalizing our findings. Finally, because the results of this study were derived from cross-sectional surveys, we cannot discuss causality. For example, people with cognitive impairment might report less social support and smaller social networks. People with no cognitive impairment possibly prefer to live in more cohesive neighborhoods. To examine causal relationships, future research including longitudinal surveys is necessary.

## Conclusions

The present population-based study found that lower levels of community-based cognitive social capital were related to a higher likelihood of cognitive impairment. Additionally, structural aspects of community social capital had a buffering role in the association between low education and cognitive impairment. These results imply that community social capital plays a protective role in the cognitive function of local residents. Our data suggest that, in addition to strategies aimed at reducing the risk of cognitive decline in individuals, building community social capital could be a valuable strategy.

## Data Availability

The datasets used and analyzed during the current study are available from the corresponding author on reasonable request.

## Abbreviations

CI: Confidence interval
MMSE: Mini-Mental State Examination
MMSE-J: Mini-Mental State Examination, Japanese version
OR: Odds ratio
